# Fine Needle Aspiration Cytology for Neck Masses in Childhood. An Illustrative Approach

**DOI:** 10.3390/diagnostics8020028

**Published:** 2018-04-22

**Authors:** Consolato Sergi, Aneesh Dhiman, Jo-Ann Gray

**Affiliations:** Department of Lab. Medicine and Pathology, University of Alberta, 8440 112 St., Edmonton, AB T6G 2B7, Canada; Aneesh.Dhiman@albertahealthservices.ca (A.D.); grayj@telus.net (J.-A.G.)

**Keywords:** cytopathology, head and neck, children

## Abstract

The primary indication of fine-needle aspiration cytology of the head and neck region is a thyroid nodule or a mass located in the cervical area or the head. Although a thyroid nodule may raise the suspicion of malignancy, less than one in 20 cases results in a carcinoma. In addition, the list of differential diagnoses is quite different according to the age of the patient. A number of benign lesions, such as branchial cysts, sialadenosis, and sialoadenitis are often seen in childhood and youth. The malignant lesions that are on the top of the list of a pediatric mass of the head and neck (H&N) region include rhabdomyosarcoma, neuroblastoma, and papillary carcinoma of the thyroid gland. This critical review of the diagnostic features of a pediatric mass of the H&N region is accompanied by panels of several cytology features that may be of help to the cytopathologist and clinician.

## 1. Introduction

In the era of transcriptomics and advancements identified at increasing pace in this 2nd decade of the 3rd millennium, an apparently simple approach with a needle, a slide, and some staining tools may be awkward. It seems that the fine needle aspiration biopsy was performed first in 1857, although several techniques and new protocols have transformed this seemingly simple procedure into a practice that is impressively diffuse worldwide. Aside from scarring in some patients, there are complications due to unnecessary surgery which is well known to many pediatricians. In adults, fine needle aspiration cytology (FNAC) has been used with great success as the primary screening test for thyroid nodules [1,2,3]. In non-thyroid nodules, FNAC has also increased rates of sensitivity and specificity in numerous studies [4]. However, such a structured approach has remained unused in the evaluation of pediatric nodules and instead, urgent analysis using scalpel and surgery is performed. This critical review of the literature highlights the information we can receive using FNAC in pediatrics and shows some illustrative cytologic features that may be extremely useful for both training or experienced cytologists and pediatricians. The present study was designed to critically review the role of FNAC and its utility in pediatric head and neck (H&N) lesions. It is not a comprehensive atlas and has no aim to fill all gaps of the H&N lesions in pediatrics, which are covered in excellent texts and books. Nevertheless, our paper emphasizes the procedure and specifies the spectrum of the H&N lesions in the pediatric age group, highlighting some studies gathered from the literature comparing cytology and histology of the H&N lesions.

## 2. Methodology

A neck mass that is resistant to antibiotics for more than four weeks should be considered the primary indication to proceed with non-thyroid FNAC. Other signs should also include cases of asymmetric benign lymphadenopathy as well as cervical lymph nodal enlargement showing unusual location, fast expansion, evidence of weight loss or loss of appetite, and night sweats. The identification of skin changes and/or a fixed/immobile mass may favor a malignant lesion and is also an indication to proceed with FNAC. An FNAC with a critical result may often be managed in our institution within a few hours planning for surgery to be performed in the same day or the day after. Nonthyroidal FNAC should be conducted while keeping in mind that the patient may be a potential surgical candidate. Other indications for an FNAC may involve an atypical presentation with systemic symptoms; the urgency made clear by the medical team or family and the willingness to diagnose the lesion before the typical 2–7 days of surgical processing. In consideration of the high percentage of papillary thyroid carcinomas in childhood and youth, FNAC should be offered as the first procedure for all pediatric thyroid nodules following an ultrasound scan showing a lesion with a diameter of 1 cm or more. In the case of pediatric thyroid nodules with lesions measuring less than 1 cm in diameter, an FNAC with a critical result processing (same day or day after or within 24 h) should be offered in the event that concerning ultrasonographic results are present. Ultrasound anomalies will include hypoechogenicity, irregularity of the margins, or increased vascularity of the lesion. FNAC is usually performed in the Otolaryngology/ear-nose-throat (ENT) clinical department or inpatient ward by specialized and well-trained personnel, including a cytopathologist, an interventional radiologist, or a (pediatric) surgeon. Topical (4% lidocaine cream) anesthesia is used with some general low sedation, and all biopsies should be performed using image guidance. In our opinion, the practice to use palpation should be discouraged or left to very palpable nodules to reduce the annual false negativity rate [5,6,7]. The procedure involves the use of a 25- to 27-gauge needle, and a range of 3 to 5 passes are performed for each targeted site, representing a single FNA procedure. If a patient has multiple targeted nodules or masses, various aspirates are usually performed at the discretion of the medical personnel. The aspiration of the material is then used for smear preparation, which includes air-dried slides stained with Diff-Quik and alcohol-fixed slides stained with the Papanicolaou stain. It is paramount to perform additional testing at the same time to avoid unnecessary delay in the diagnosis. In fact, residual material is typically used for ThinPrep processing (Hologic Inc., Marlborough, MA, USA), microbiology, and molecular biology studies. To confirm whether the material is adequate in a lymph node, a small drop of the aspirate is placed onto the preparation slides for immediate assessment (also technically labelled ‘rapid on-site evaluation’), while the remainder is rinsed in a cell preservative. Typically, 10 million cells are considered adequate for cytopathology assessment, which requires 2–3 passes indeed. 

The cell block preparation is routinely performed in our institution and not only in case of ambiguous interpretation of the aspirates. It is also important to advise the lab technician to stain some tissue sections of the cell block with hematoxylin-eosin and keep some unstained tissue sections for histochemical (special) stains, immunohistochemical stains, and further ancillary testing as required. The cytology aspirates are interpreted initially by a cytotechnologist and subsequently by a pediatric pathologist with an intra-institutional cytopathology consultation adjured by a cytopathologist. FNAC cases receive diagnoses with an adequacy interpretation (unsatisfactory, satisfactory), a primary interpretation (nondiagnostic, negative for malignant cells, atypical cells present, suspicious for cancerous cells, or positive for malignant cells), and a free text explanatory diagnosis. The category ‘less than optimal’ is not often used in our institutions to avoid non-categorical decisions.

It is also advisable to perform a yearly outcome rating using spreadsheet-based correlation to surgical histopathology and clinical follow-up. In the case of clinical monitoring, laboratory values and clinical presentation are part of a 6-month–1-year follow-up, while surgical histopathology, which is based on either an incisional surgical biopsy or an excisional surgical biopsy, is considered the reference standard in the diagnosis of tumors of the H&N region. In the setting of a quality assurance program, true positives are cytopathologic results that warrant surgical treatment and are confirmed as such using surgical histopathologic biopsy. True negatives are cytopathologic results that did not designate a need for surgery and are established as such using the surgical histopathologic biopsy. Clinical true negatives are negative conditions on cytology that resolve or did not progress without surgical intervention. In case of false positives cytology, cytopathology results are positive, but there would have been no need for surgical treatment, while false negatives are unremarkable cytopathologic results without a need for surgery, although a surgical intervention would have been the right option. To avoid bias in any cytology-pathology correlation quality assurance program, it is prudent that pathologists interpreting surgical histopathology slides are distinct from cytopathologists interpreting the FNAC. Some infective or non-malignant conditions, such as atypical mycobacterial infection, cervicofacial abscess, or lymphatic malformations, may require surgical diagnosis or treatment but may also appropriately be managed by medical therapy. If the appropriate medical treatment is initiated and results efficacious and efficient, the result is labeled as an actual clinical positive. The 2015 Standards for Reporting of Diagnostic Accuracy (STARD) guideline for reporting diagnostic accuracy studies should be used [8].

In the setting of procedure complications, care should be taken when considering the side effects of FNAC, e.g., infection and damage to nearby structures. By reviewing the literature, we found a limited core of evidence for such side effects if a safe procedure is applied. In particular, an honest and straightforward report provided by Chen et al. [9], who reported a 19-year-old girl with a painful goiter, which became more painful after fine needle aspiration (FNA). The patient’s second FNAC revealed only many neutrophils and an antibiotic treatment improved the pain, but the goiter persisted. The third FNA revealed cytology of papillary carcinoma cells and the total thyroidectomy showed ischemic necrosis with neutrophilic aggregation around the needle track other than a papillary thyroid carcinoma. This report reveals the possibility of missing malignant cells in a background of an infection following an improper FNA. Secondary infection and ischemic necrosis may indeed occur after FNA, and aseptic procedures are necessary to prevent bacteria from seeding into the thyroid gland. Disinfection of the FNAC site and experienced personnel are mandatory for laboratories that harbor periodic accreditation by the College of the American Pathologists (CAP) operating in healthcare institutions in United States of America and Canada. Thus, side effects may occur following an FNA procedure. Nevertheless, we consider that FNAC in children and adolescents is accurate, safe, and well-accepted in a wide range of lesions of the head and neck regions. A CAP accreditation or a similar accreditation outside of North America should be requested by all pediatricians and parents of the affected child.

## 3. Diagnostics

In most of the cases, FNAC of the H&N region is performed to investigate clinically suspicious lymphadenopathy, and the primary differential diagnoses include congenital anomaly, reactive/infectious lymphadenopathy, lymphoma, and metastatic disease, but also thyroid gland disease and salivary gland disease.

### 3.1. Congenital Lesion

The age is also particularly important to keep in mind, and 75% of branchial cysts occur in 20–40 year-old patients [10,11,12,13,14]. Benign squamous-lined cysts are usually characterized by abundant inflammatory cells (neutrophils), with few unremarkable squamous epithelial cells with bland nuclear features, and cholesterol crystals, while a large number of squamous cells with or without occasional nuclear hyperchromasia, nuclear membrane irregularity, and high nucleus to cytoplasm ratio portend for a malignant diagnosis.

### 3.2. Reactive/Infectious Lymphadenopathy and Malignant Lymphoma

In about 2/3 of cases, de novo lymphadenopathies of the H&N region are benign, and even a history of malignancy present in a child with a de novo lymphadenopathy will have a rate of benignancy probably approaching half of the cases [15,16]. Moreover, additional surgical procedures have been avoided in up to 61% of cases in one review [17]. The cytopathological diagnosis of reactive is mostly accurate in children and youth with less in 1 out of 20 (~5%) subsequent (false-negative) malignant diagnoses [2,18]. Cytologic features of reactive lymphadenopathy include polymorphic lymphoid cells showing a clear-cut sequence of maturation, prominent reactive cells arising from germinal centers, the presence of tingible body macrophages, the lack of a monomorphic lymphoid population and the simultaneous presence of a variegated (non-homogeneous) cell population smear, and the lack of a subpopulation (even minimal) of large, irregular lymphoid cells potentially indicative of Hodgkin’s lymphoma, anaplastic T-cell lymphoma, large B-cell lymphoma). Necrotizing granulomata are often characteristic of underlying tuberculous infection (particularly systemic), although they may also be found in fungal infection and other acid-fast bacilli (AFB) (e.g., mycobacteria causing scrofula). In a child, the finding of granulomata may suggest non-tuberculous AFB, especially in a child, and cat-scratch disease should be considered in this age group. Granulomas without evidence of necrosis are suggestive of sarcoidosis, toxoplasmosis, or foreign-body giant cell reaction. In all cases, a thorough microbiological investigation is paramount (e.g., *Bartonella henselae* for cat-scratch disease). The correlation between clinical and laboratory findings using electronic medical charts or liaising with the clinician is essential. In fact, serological tests may be useful in a subset of cases for differentiating systemic lupus erythematosus lymphadenitis from Kikuchi–Fujimoto’s disease, which is histiocytic necrotizing lymphadenitis. Finally, it is important not to forget that a florid granulomatous pattern can mislead the cytopathologist disguising both Hodgkin’s lymphoma and Non-Hodgkin’s lymphoma.

### 3.3. Rhabdomyosarcoma and Neuroblastoma

**Figure 1:**
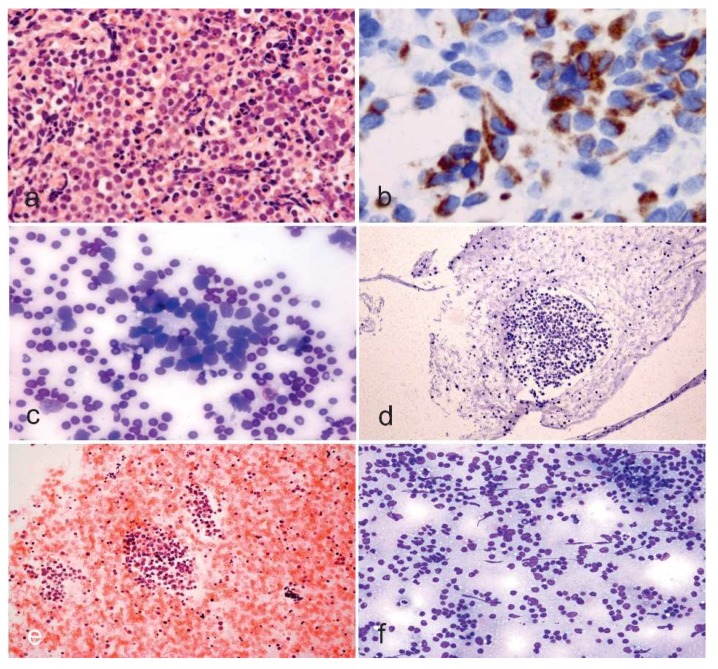
(**a**,**b**) Rhabdomyosarcoma cytology in cell block (Papanicolaou, ×200) and smears fixed and immunostained with the antibody anti-desmin (Avidin-Biotin-Complex immunostaining, ×630). (**c**,**d**) Neuroblastoma cytology in smears and cell block showing hyperchromatic cells with high nucleus to cytoplasm ratio (Diff-Quik, ×100 and ×100, **c** and **d**, respectively). (**e**,**f**) Reactive changes of soft tissue showing a mixture of cells with variable morphology (cell block and smears stained with Papanicolaou and Diff-Quik, ×100 and ×100, **e** and **f**, respectively).

In a pediatric setting, the most fearful malignant diagnoses in the H&N region, apart from malignant lymphoma, are rhabdomyosarcoma and neuroblastoma. Rhabdomyosarcoma is considered to harbor three main subtypes, including embryonal, which is the most frequent type, the alveolar, and the pleomorphic, which most commonly seen in adults. Rhabdomyosarcoma occurs very often in the head and neck region and the extremities, with the trunk reserved for the third location. Cytomorphology features of the embryonal rhabdomyosarcoma include a variability of patterns including round to spindle-shaped cells, some pleomorphism (different from the marked pleomorphism of the pleomorphic type of the rhabdomyosarcoma of the adult), a variable number of rhabdomyoblasts may be the distinctive clue for the cytopathologist, and, occasionally, inclusion-like cytoplasmic condensation, which is also known as myogenic differentiation [18]. Rhabdomyoblastic differentiation is characterized by elongated, strap- or tadpole-shaped cytoplasm, and nuclear eccentricity. The nuclear diagnostic clues also rely on dense hyperchromatic chromatin and nuclear membrane irregularity (Figure 1a,b). The alveolar rhabdomyosarcoma is distinctively identified by larger, uniformly round to variably polygonal cells with an increased number of mitotic figures in the background with scattered rhabdomyoblasts and giant cells with wreath-like nuclei. Metastatic neuroblastoma to the neck is quite rare in adulthood, but it is a principal differential diagnosis in infants and children, especially in infants. Although most neuroblastomas arise in the adrenal gland or the paravertebral sympathetic chain bilaterally, this pediatric tumor can occur anywhere along the sympathetic chain. Cytomorphology of neuroblastoma may be entirely different according to the degree of ganglionic differentiation of the neuroblastoma. These features include a fibrillary matrix, dyshesive small undifferentiated cells, sparse Homer–Wright rosettes, a nuclear pattern labeled as “salt and pepper” for the variability of the granularity of the nuclear chromatin, and, occasionally, ganglion-like cells (Figure 1c,d). In rare occasions, necrotic debris and dysmorphic calcifications are seen [18]. It is paramount to distinguish reactive/reparative changes from the diagnoses mentioned above [19] (Figure 1e,f). A spectrum of appearance needs to be kept in mind in case of benign fibrous proliferation. The retention of a low nucleus to cytoplasm ratio is crucial in case of prior radiation where cells may be large, showing irregularity of the nuclei, multinucleation, all features that may be quite worrisome. In fact, the resolution may decrease the inflammatory background, making the diagnosis particularly distressful for an inexperienced pathologist. Moreover, the myofibroblasts that occur in benign fibrous proliferations may become slender with less conspicuous nucleoli during the healing process. In all cases, the identification of pleomorphism, nuclear hyperchromasia, high nucleus to cytoplasm ratio, and atypical mitoses suggest malignancy that needs to be surgically acted upon as soon as possible. 

### 3.4. Thyroid Gland Disease

The introduction of synoptic reports has been crucial in improving healthcare worldwide, allowing a common platform for adequately assessing patients with neoplastic disease. Thyroid cases receive an adequacy interpretation, a first interpretation using “The Bethesda System for Reporting Thyroid Cytopathology” (TBSRTC), and a free text explanatory diagnosis with a comment according to the pathologist or cytopathologist [2]. It is also encouraged that the cytopathologist or the pathologist liaise with the clinical team in case an FNAC may indicate suspicious malignant cells or some distinct cancerous cells. TBSRTC categories include insufficient for diagnosis, benign, atypical cells of undetermined significance, suspicious for a follicular neoplasm, suspicious for a Hürthle cell neoplasm, suspicious for malignancy (papillary carcinoma, medullary carcinoma, lymphoma, metastatic tumor, other), and malignant. Despite iodine supplementation, multinodular goiter (MNG) occurs in North America. MNG is a common disorder of the thyroid gland with multiple nodules [18]. The macrofollicular pattern of a follicular neoplasm is indistinguishable from MNG Cytologically, a benign follicular nodule shows mainly macrofollicles, low/moderate cellularity, cohesive follicular cells with uniformity and even spacing, coarse chromatin, as well as colloid, which may be copious. The differential diagnosis of benign follicular nodules includes suspicious follicular nodule, suspicious Hürthle cell nodule, and papillary carcinoma. In some benign nodules, focal cytologic atypia may be present and becomes a diagnostic challenge. Of note, Hürthle cell metaplasia is often seen in MNG and the cells may show at places marked nuclear atypia. Hashimoto or chronic lymphocytic thyroiditis is characterized by a mixed population of lymphocytes, tingible-body macrophages, dendritic-lymphocytic clusters, Hürthle cells and scant colloid. Hyperplastic Hürthle cells nodules may occur in Hashimoto thyroiditis mimicking a Hürthle cell tumor [18]. The differential diagnosis of Hashimoto thyroiditis includes a reactive lymph node, MNG with predominant Hürthle cell change, primary malignant lymphoma of the thyroid gland, Hürthle cell neoplasm, and papillary thyroid carcinoma. A subacute thyroiditis and Riedel disease are not a common event in pediatric age or youth. The category of suspicious for a follicular neoplasm has some cytologic features including marked cellularity, scant colloid, predominant microfollicles or epithelial trabeculae, and enlarged, crowded follicular cells (differently from the uniformity and even spacing of the benign lesions) [18]. In a pediatric setting, a suspicious for a follicular neoplasm poses a differential diagnosis of a benign follicular nodule, papillary thyroid carcinoma, and parathyroid adenoma or carcinoma. Other malignant masses have other distinguishing features in childhood and youth. A suspicious for Hürthle cell neoplasm diagnosis has cytologic features showing an almost complete cell population of Hürthle cells, usually dyshesive cells with prominent nucleolus, and pseudopsammoma bodies. Cytological features of papillary thyroid carcinoma include sheets, microfollicles, and papillae, although the nuclear changes need to be searched carefully. The nuclear changes include grooves, pseudoinclusions, “powdery” chromatin, and thickened and irregular nuclear membrane. There is also nuclear crowding or molding. Cells show a variable amount of cytoplasm and psammoma bodies may be present. The differential diagnosis of papillary carcinoma includes a benign follicular nodule, a follicular neoplasm, Hashimoto thyroiditis, radiotherapy effect, and a hyalinizing trabecular tumor. Although the anaplastic carcinoma does not play any role in childhood or youth, an important entity to keep in mind is the medullary carcinoma. Cytologically, this endocrine parafollicular cells derived tumor shows numerous isolated cells, loose aggregates of cells with epithelioid, plasmacytoid, or spindle-shaped morphology, nuclear changes (rounding or elongation, granularity of the chromatin, inconspicuous nucleolus, pseudoinclusions, and multiple nuclei), cytoplasmic changes (red granules) on Diff-Quik stained preparations, and amyloid. Although rare, two primary malignant lymphomas of the thyroid gland need to be kept in mind and include the diffuse large B cell lymphoma and the extra-nodal marginal zone B-cell lymphoma.

### 3.5. Salivary Gland Disease

**Figure 2:**
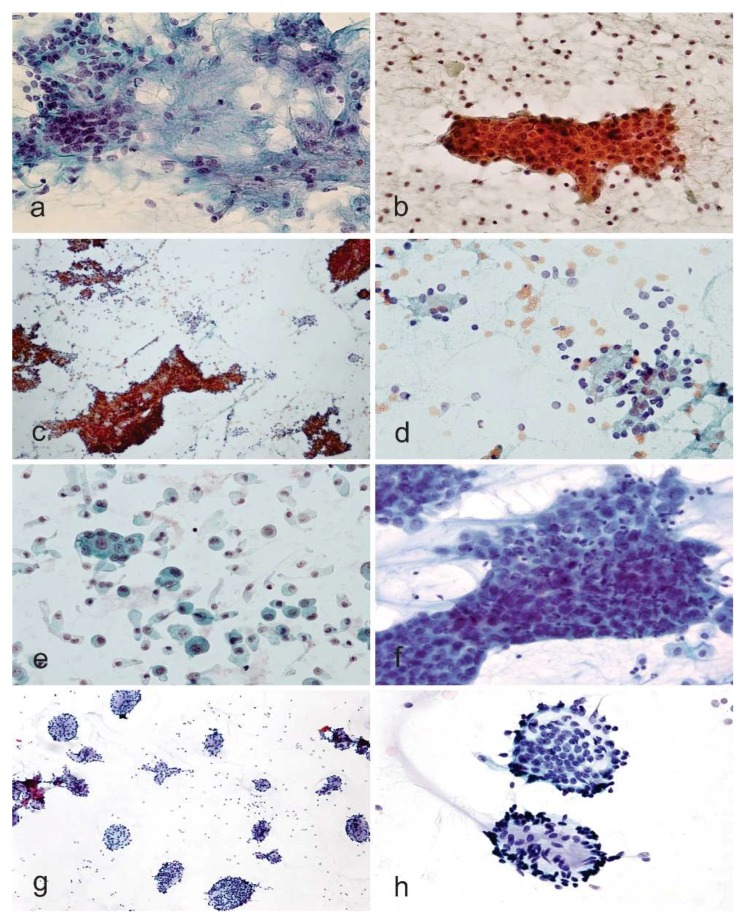
(**a**,**b**) Pleomorphic adenoma and Warthin tumor (Papanicolaou stain, ×100 and ×100, **a** and **b**, respectively). (**c**,**d**) Acinic cell carcinoma (Papanicolaou stain, ×40 and ×100, **c** and **d**, respectively). (**e**,**f**) Mucoepidermoid carcinoma (Papanicolaou stain, ×400 and Papanicolaou stain, ×400, **e** and **f**, respectively). The mucin in the background and the nuclear detail are supportive of a diagnosis of mucoepidermoid carcinoma. (**g**,**h**) Adenoid cystic carcinoma (Diff-Quik, ×100 and ×100, **g** and **h**, respectively). For the details of the single salivary gland tumors, please refer to the text.

Salivary gland lesions are rare in children, although an increase of salivary gland disease in adolescence and youth has been reported recently [20,21,22]. Previous research has shown that salivary gland tumors are rare in the young population, but the incidence of all primary salivary gland carcinomas increased with increasing patient age, particularly in patients younger than 30 years. Lesions of the major cephalic salivary glands, with the exception of mumps and cytomegaly, are unusual in children and adolescents, but these lesions may give rise to some different tentative diagnoses [23,24,25]. Since malignant salivary gland tumors are relatively more frequently recognized in younger patients, a safe approach is advisable, and intradepartmental consultation is crucial. Cytologic diagnoses of malignant tumors are confirmed histologically in 93% of cases, while benign tumor diagnoses are confirmed on histology in 95% of cases. Inflammatory lesions are confirmed on histology in 73%, while benign salivary gland tissue is ascertained as such histologically in 18% of cases [26]. Non-rare malignant salivary gland tumors in childhood and youth are a mucoepidermoid carcinoma, adenoid cystic carcinoma, acinic cell carcinoma, malignant lymphomas, and metastatic tumors from a tumor of the H&N region or paraganglia (e.g., neuroblastoma). Benign neoplasms include pleomorphic adenoma and Warthin’s tumor (Figure 2a,b). In childhood, 80–90% of all malignant salivary gland tumors are constituted by mucoepidermoid carcinomas, adenoid-cystic carcinomas, and acinic cell carcinomas.

In adults, the corresponding figure is only 45% [23]. Despite the low incidence of 15–25% of malignant neoplasms for adults identified in 1969 [27], in a study carried out on 2632 patients following teams and investigations reported a significantly higher relative proportion in young patients [23]. Acinic cell carcinoma is characterized by a hypercellular aspirate with a clean background with tumor cells seen in disorganized clusters with loss of round groupings and lack of an associated ductal epithelium and on higher magnification cells show some uniformity resembling normal serous acinar cells with the cytoplasm being foamy or bubbly and harboring fine dark granules (Figure 2c,d) [28,29]. The examination of the background highlights many naked nuclei. Apart from clear cell tumors, mainly low-grade mucoepidermoid carcinoma and epithelial-myoepithelial carcinoma), oncocytic tumors, sialadenosis, and normal salivary gland tissue need to be kept in the differential diagnosis list. Smears of mucoepidermoid carcinoma are usually low in cellularity with a particularly striking dirty background of mucin and debris. In the smears, the presence of scattered cell clusters of intermediate cells with overlapping epithelial groups, some mucin-coated cells (goblet-cells-like), and few squamous epithelial cells are particularly evident features for the diagnosis of mucoepidermoid carcinoma (Figure 2e,f). However, both components may be quite bland or missing in one or all slides. According to the grade of differentiation, nuclear features may vary from bland to hyperchromasia with a high nucleus to cytoplasm ratio, although this tumor is usually low-grade in childhood and adolescence. Some potential pitfalls in diagnosing the mucoepidermoid carcinoma are some cystic nature of these tumors, which may harvest only hypocellular (even acellular in some cases) mucoid material [30]. Moreover, extracellular mucin is typically copious, mimicking the fibrillary stroma seen in pleomorphic adenomas, although the mucin of pleomorphic adenomas stains less intensely and has no fibrillary pattern. Finally, squamous metaplasia is not only characteristic of mucoepidermoid carcinoma but may be found in other tumors, such as pleomorphic adenoma and Warthin’s tumor (Figure 2a,b). The adenoid cystic carcinoma features are large globules of extracellular matrix with or without surrounding basaloid cells. In FNAs with a predominance of basaloid tumor cells, both benign and malignant salivary gland tumors of epithelial–myoepithelial differentiation should be considered in the differential diagnosis (Figure 2g,h) [28,29]. If TBSRTC has received a warming acceptance in many countries, the Milan System for Reporting Salivary Gland Cytology (MSRSGC) seems that will have similar success in the coming years [31,32,33,34,35]. The MSRSGC is an evidence-based tiered classification system that comprises six diagnostic categories associated with an average risk of malignancy (ROM) and clinical management strategies (Table 1). It is expected that the MSRSGC will improve communication between the cytopathologist and the treating pediatrician, facilitating cytologic-histologic correlation, and lead to overall improved patient care.

## 4. Study Comparison

FNAC is now being considered as a valuable diagnostic aid in many children’s hospitals across continents, not only in North America. In most studies, FNAC reveals good aspects, showing the early availability of results, its simplicity, minimal trauma or injury, and the low rate of complications. Ancillary techniques, including flow cytometry, cytogenetics, immunohistochemistry on the cell block, and electron microscopy can be easily applied for the characterization of tumors [20,21,37,38]. Furthermore, FNA does not usually need heavy sedation, and general anesthesia is not necessary. FNAC of the H&N region is a well-accepted technique that has high specificity in most studies [21]. In Table 2, a comparison of four studies is provided.

## 5. Limitations

Most of the studies have several limitations that are inherent in their retrospective nature. First, the lack of standardization of the enrolled patients is reflective of both the diversity of pediatric nodules and the variable diagnostic and treatment approaches of different clinical and surgical teams [17]. In fact, there is often a significant variation in the use, duration, and timing of antibiotic treatment, and the accessibility of the clinical and surgical services was also variable and change over time was present in most of the retrospective studies. The lack of universal follow-up is also an essential drawback of numerous FNAC studies. The importance of the monitoring may be enshrined for all patients with negative FNAC results that need to be instructed to follow up if the mass persists, increases in size, or any concerns remain.

## 6. Conclusions

The potential avoidance of surgery with associated scarring, early and late septic and non-septic surgical complications, general anesthetic risk, recovery time, and significant expense bills have all been heralded as benefits of FNAC, especially given the high prevalence of non-neoplastic pediatric tumor and masses of the H&N region. FNAC shows an impressive diagnostic accuracy in numerous studies. FNAC is supported by an active lab safety profile and carries a very limited number of drawbacks considering that in the case of a dubious cytology result, a surgery can be planned in a very short time identifying FNAC as a useful triage procedure. In conclusion, FNAC is a safe, well-tolerated, and accurate tool for diagnosing pediatric masses of thyroid and non-thyroid origin in childhood and youth. FNAC plays a vital role in giving comfort to obviate the need for unnecessary immediate surgery in benign nodules (congenital/reactive/inflammatory) and plan a primary surgical intervention in malignant lesions. In consideration of significant health care changes worldwide, we would like to strongly emphasize that when such a triage of pediatric masses of the H&N region is handled, there is a potential decrease in both surgical morbidity and health care costs.

## Figures and Tables

**Table 1:** Milan System for Reporting Salivary Gland Cytology (adapted from [36]).

Category significance
Category I => Nondiagnostic, harboring a ROM of 25%
Category II => Nonneoplastic, harboring a ROM of 10%
Category III => Atypia of undetermined significance, harboring an estimated ROM of 20%
Category IV => Neoplasm
Subcategory IVA => Benign, harboring a ROM of <5%
Subcategory IVB => Salivary gland neoplasm of uncertain malignant potential, harboring a ROM of 35%
Category V => Suspicious for malignancy, harboring a ROM of 60%
Category VI => Malignant, harboring a ROM of 90%

Notes: ROM, risk of malignancy.

**Table 2:** Study Comparison (adapted from [21]).

Study	Rapkiewicz [37]	Jain [20]	Handa [38]	Mittra [21]
Topics	H&N lesions	H&N lesions	Cervical LNs	H&N lesions
Cases	85	748	692 (584 LNs)	100
Age group	0–18 years	0–12 years	0–14 years	0–15 years
Adequacy	N.A.	94%	93.4%	93%
Common site	LN (69.4%)	LN (81%)	LN (84.3%)	LN (87%)
Benign	83%	98.5%	98.5%	88.2%
Malignant	17%	1.5%	1.5%	11.8%

Notes: H&N, Head and Neck; LN, lymph nodes; N.A., not available.
